# Development and Validation of a Measure of Parents’ Psychological Need Satisfaction and Frustration When Involved in Their Child’s Schooling

**DOI:** 10.1177/10731911251348769

**Published:** 2025-07-13

**Authors:** Catherine F. Ratelle, Geneviève Boisclair Châteauvert, Jiseul Sophia Ahn, Jolene van der Kaap-Deeder, André Plamondon, Julien Bureau, David Litalien

**Affiliations:** 1 Université Laval, Quebec, Canada; 2 Norwegian University of Science and Technology, Trondheim, NorwayJiseul Sophia Ahn is now affiliated with Université de Montréal

**Keywords:** psychological needs, parenting, school involvement, autonomy, competence, relatedness, primary school

## Abstract

This study evaluated the psychometric qualities of the adaptation of the Basic Psychological Need Satisfaction and Frustration Scale (BPNSFS) to the context of parents’ school involvement (PSI). The BPNSFS-PSI assesses the satisfaction and frustration of parents’ needs for autonomy, competence, and relatedness when involved in their child’s schooling. Two samples of parents whose child attended primary school in Grades 1 to 6 (Sample 1; *N* = 1,017, 63% mothers) or in Grades 1 and 2 (Sample 2; *N* = 1,448; 74% mothers), filled out an online questionnaire. The BPNSFS-PSI showed adequate internal consistency, structural validity via a bifactor exploratory structural equation modeling measurement model, and construct validity via correlations with parents’ vitality and satisfaction with their family life. Measurement invariance was supported across samples, parent gender, and child school cycle. Together, these findings demonstrate the reliability and validity of the BPNSFS-PSI in both mothers and fathers, supporting its use for measuring parents’ needs in the context of their school involvement. The development and validation of this scale will encourage further research by providing a reliable tool to assess parents’ satisfaction and frustration with their basic psychological needs in school involvement.

Parental involvement in schooling, while a challenge and source of conflict, is an important lever for child development and can vary in quality and intensity, with some forms being more optimal (e.g., autonomy supportive involvement; Grolnick & Pomerantz, 2022) than others (e.g., helicopter parenting; Padilla-Walker & Nelson, 2012). According to self-determination theory (SDT; Ryan & Deci, 2017), the most positive child outcomes arise when parental involvement is highly autonomy supportive, structuring, and warm. This high-quality involvement provides children with the resources to self-actualize, regulate their behaviors, and thrive (Grolnick & Pomerantz, 2022; Soenens et al., 2019). Given the benefits of high-quality parental involvement in children’s schooling, it is crucial to understand how to facilitate such practices. Here, we focus on parent-related factors essential for high-quality involvement, namely parents’ psychological needs for autonomy, competence, and relatedness, as proposed by SDT (Ryan & Deci, 2017). Research showed that parents’ autonomous motivation to engage in their child’s homework benefits both the child’s achievement and parental involvement (Green et al., 2007; Grolnick, 2015; Moè et al., 2020). Additionally, parents who feel more competent provide better support for homework (Katz et al., 2011), while those with limited formal education are less involved (Durand, 2010). Furthermore, mothers who feel connected to their child during discussions about school are more autonomy supportive (Clark & Ladd, 2000). Thus, the quality of parents’ school involvement (PSI) depends on how well their own needs are met.

Despite this indirect evidence for the role of parents’ needs in their school involvement, no extant measure assesses these needs within this context—existing measures are either general or contextualized to other contexts. This study, therefore, aimed to develop and evaluate the psychometric qualities of the Basic Psychological Need Satisfaction and Frustration Scale-Parents’ School Involvement (BPNSFS-PSI).

## Defining Psychological Needs

SDT posits that all human beings have three innate psychological needs whose satisfaction is fundamental to individuals’ development, thriving, and well-being (Ryan & Deci, 2017; Ryan et al., 2021). The need for *autonomy* refers to acting volitionally and with a sense of choice. In the domain of school involvement, autonomy satisfaction manifests when parents engage in their child’s schooling willingly, with interest, and because they value doing so, whereas autonomy frustration can be displayed through a sense of obligation or coercion toward their involvement. Second is the need for *competence*, which is characterized by interacting with one’s environment in an efficient way and perceiving that one’s actions are producing expected and desired consequences. In the current context, competence satisfaction is displayed when parents feel able to support and help their child with academic tasks (e.g., homework, studying). In contrast, competence frustration occurs when parents doubt their ability to effectively engage in their child’s schooling or do not understand how to go about it. Finally, the need for *relatedness* refers to the need to be accepted by significant individuals and to develop meaningful reciprocal relationships with them. Its satisfaction is observed in the current context when parents experience a sense of connection with their child when engaging in school-based interactions, whereas its frustration during their school involvement may be observed when parents experience interpersonal friction and distance with the child or other meaningful individuals, like their teacher.

According to SDT, individuals engage more positively with others and experience optimal functioning when their psychological needs are satisfied rather than frustrated (see Ryan & Deci, 2017; Vansteenkiste & Ryan, 2013; Vansteenkiste et al., 2020). This concurs with past conclusions that parents more adequately support their child when their psychological needs are met (Grolnick, 2003; Ryan & Deci, 2017).

### Parents’ Psychological Needs

Abundant research offered support for the importance of these psychological needs in several contexts such as work, sports, leisure, and education (Ryan & Deci, 2017). Although parental need satisfaction is expected to relate to many aspects of their children’s lives, as well as their own, we identified only two studies that examined parents’ need satisfaction and frustration in the context of parental involvement in their child’s schooling (Katz et al., 2011; Moè et al., 2020). In fact, most studies that examined parents’ psychological needs did so in general contexts (Costa et al., 2019; de Haan et al., 2013; Mabbe et al., 2018; Slobodin et al., 2020; van der Kaap-Deeder et al., 2015, 2019) or in specific domains such as parenting an infant (i.e., as emerging parenthood; Brenning et al., 2019). This body of research supports the relevance of studying parental needs, but their application to school involvement is more distal. Indeed, children stay in school for around 15 years during the most formative time, which makes it vital to study how parents are involved in their child’s schooling. Some studies did consider the context of PSI, but only examined one of the three needs, and most did not capture need frustration. For instance, the work of Katz et al. (2011) examined parents’ competence satisfaction in their school involvement, which was linked to their motivations, attitudes, and self-reported behaviors. Relatedly, the literature on parental self-efficacy—which is theoretically related to competence satisfaction—examined parents’ perceived competence in children’s school-related activities (Glatz & Buchanan, 2015), in managing children’s transition to school (Giallo et al., 2008), or children’s internet use (Hsieh et al., 2017). Using the frustration items of the BPNSFS, Moè et al. (2020) examined parents’ overall need for frustration during homework, which they found to predict homework stress and controlling behaviors.

In general, studies on parents’ psychological needs showed that need satisfaction was linked to greater parental autonomy support, which predicted their child’s need satisfaction, and that need frustration led to greater parental control and child need frustration (Costa et al., 2019; Moè et al., 2020). Similar positive outcomes associated with need satisfaction (and conversely for need frustration) were obtained in intensive longitudinal studies with parents of adolescents (e.g., Mabbe et al., 2018) and elementary school children (van der Kaap-Deeder et al., 2019), but in which parents’ psychological needs were assessed in general, not contextualized to their school involvement.

## Assessing Parental Needs

Studying parents’ psychological needs can be done using several methodological approaches such as interviews, observation, or surveys. Research on psychological needs in general frequently resorted to questionnaire measures, with the *Basic Psychological Need Satisfaction and Need Frustration Scale* (BPNSFS; Chen et al., 2015; van der Kaap-Deeder et al., 2020) being the most popular and commonly used measure. Among the other scales that assess the psychological needs identified by SDT is the *Psychological Need Satisfaction Scale* (Costa et al., 2015), which has been adapted to the parenting context (Costa et al., 2019), although it only assesses need satisfaction and has not been contextualized to school involvement. In the parenting literature, the *Parenting Stress Index* (Abidin, 1990; Jones & Prinz, 2005) has been used to assess constructs akin to psychological need satisfaction (i.e., sense of competence, attachment, and role-restriction scales; de Haan et al., 2013). Other measures focus specifically on one need, and mostly its satisfaction: (a) parental competence (or self-efficacy) with scales such as the *Parents’ Perceived Competence Scale* (Johnston & Mash, 1989), the Self-Efficacy for *Parenting Tasks Index* (Coleman & Karraker, 2000), the *Parents’ Self-Efficacy in Managing Transition to School Scale* (Giallo et al., 2008), l’*Échelle Globale du Sentiment de Compétence Parentale* (Global Parental Competence Scale; Meunier & Roskam, 2009), the *Parental Self-Efficacy Scale* (Ballenski & Cook, 1982), the *Parental Self-Efficacy Scale* (Freedman-Doan et al., 1993), or the *Parents’ Perceived Self-Efficacy to Manage Children’s Internet Use Scale* (Hsieh et al., 2017); (b) parental relatedness (or attachment/relationship quality) with the *Inventory of Parent and Peer Attachment* (Armsden & Greenberg, 1987) and the *Conflict Behavior Questionnaire* (Prinz et al., 1979); and (c) parental autonomy via the role-restriction scales of the *Parenting Stress Index* (Abidin, 1990; Jones & Prinz, 2005) used by de Haan et al. (2013).

Apart from the BPNSFS, these measures do not include subscales assessing the frustration of these needs. Assessing need frustration can be useful to predict negative outcomes, as per a dual process model (e.g., Jang et al., 2016). The studies that used the BPNSFS assessed satisfaction and frustration of all three needs and often considered the subscales separately. Most, if not all, studies with the BPNSFS aggregated the scores in the three needs to create a global factor of parental needs satisfaction and frustration. Hence, researchers wanting to assess both the satisfaction and frustration of parents’ psychological needs for autonomy, competence, and relatedness do well to use the BPNSFS, which has been adapted to a plethora of domains and validated in many languages (see van der Kaap-Deeder et al., 2020). It has been used in a parenting context for assessing parental needs in their general interactions with their child (only satisfaction items; Schrooyen et al., 2021), toward caring for their infant child (Brenning et al., 2019), or in supervising their child’s homework (only frustration items; Moè et al., 2020). Contextualizing this measure to the context of school involvement will allow a better prediction of parenting behaviors in that specific context, as shown with parental autonomy support (van der Kaap-Deeder et al., 2015). Hence, to be able to assess all three needs, both with respect to their satisfaction and frustration, in the context of parents’ involvement in their child’s schooling, we need to adapt the BPNSFS to contextualize it more closely to school involvement. While it has partially been done with respect to frustration items, and specifically for homework supervision (Moè et al., 2020), a more encompassing adaptation—that covers a wide range of parents’ involvement in their child’s schooling—is still in the waiting. Thus far, studies on parental needs that assessed all three needs for both their satisfaction and frustration (e.g., Mabbe et al., 2018) were not contextualized to such parenting and used a restricted set of items because of their research design (diary study).

## The Present Study

The goal of this study was to adapt the BPNSFS to the context of PSI (BPNSFS-PSI), to allow researchers to adequately measure how parents’ needs for autonomy, competence, and relatedness are satisfied/frustrated when they engage in their child’s schooling. Items from two existing measures were used to create this adaptation: (a) the French version of the BPNSFS (Chevrier & Lannegrand, 2021), which assesses individuals’ need satisfaction and frustration in general, and (b) the BPNSFS contextualized to parenting domains such as infant caregiving (Brenning et al., 2019). To ascertain the psychometric qualities of the BPNSFS-PSI, we sought to validate it in two large independent samples of parents whose child attends primary school, a critical period for parental school involvement (Carlson et al., 2019).

The reliability of the BPNSFS-PSI will be estimated using two types of evidence, namely the scales’ internal consistency and the test-retest reliability (AERA, APA, & NCME, 2014; Furr, 2020; Kline, 2020). Once the precision of the scale is demonstrated, its validity will then be examined. Because the current measure is an adaptation of an existing measure (BPNSFS) based on SDT (i.e., supporting its content validity), we focus here on evidence based on its internal structure (structural validity), response process (generalizability), and relation to other variables (predictive validity) (AERA, APA, & NCME, 2014; Furr, 2020; John & Benet-Martinez, 2014; Kline, 2020).

Structural validity is tested by estimating and comparing measurement models with distinct parameterizations. Since needs are multidimensional constructs, confirmatory factor analysis (CFA) and exploratory structural equation modeling (ESEM; Marsh et al., 2014) were conducted. ESEM is an alternative to CFA that does not rely on the assumption that cross-loadings are exactly zero, as CFA does, thereby making ESEM less sensitive to slight deviations from this untenable assumption. ESEM often offers a superior fit when modeling multidimensional constructs in general, and specifically for the BPNSFS (e.g., Tóth-Király et al., 2018), which we expected to replicate. Based on the work by Tóth-Király et al. (2018) that aimed to validate the general BPNSFS in Hungarian adults, we estimated 17 measurement models whose factor parameterization either constrained cross-loadings to 0 (CFA) or allowed each item to load on each factor (ESEM). Models varied on: (a) their number of latent factors, where all 24 items could load on a single factor (representing need fulfillment; Model 1), two factors (representing need satisfaction, need frustration; in CFA [Model 2] and ESEM [Model 3]), three factors (autonomy, competence, relatedness; in CFA [Model 4] and ESEM [Model 5]), or six factors (autonomy satisfaction, autonomy frustration, competence satisfaction, competence frustration, relatedness satisfaction, relatedness frustration; in CFA [Model 6] and ESEM [Model 7]); and (b) their hierarchical structure, where items could load on specific factors only or on specific factors and global factors (bifactor model; Morin et al., 2016). Because there could be a hierarchical factor for both need satisfaction or need frustration or a single global factor (where the satisfaction items would load positively, and frustration items would load negatively), both versions of the bifactor model were estimated. Hence, 10 bifactor models were estimated using the same strategy as for nonhierarchical models presented above: (a) two specific factors and one global factor (in CFA [Model 8] and ESEM [Model 9]); (b) three specific factors with one global factor (in CFA [Model 10] and ESEM [Model 11]) and with two global factors (in CFA [Model 12] and ESEM [Model 13]); (c) six specific factors with one global factor (in CFA [Model 14] and ESEM [Model 15]) and with two global factors (in CFA [Model 16] and ESEM [Model 17]). We expected to replicate Tóth-Király et al.’s (2018) finding where a superior fit was obtained with a bifactor ESEM model (Morin et al., 2016) when estimating the factorial structure of the BPNSFS.

Once the structural validity is demonstrated, the retained model will be used to test the generalizability (a) across samples, (b) across mothers and fathers, (c) as a function of the child’s sex, and (d) as a function of the child’s education level. To do so, invariance analyses will be conducted across subsamples, comparing configural, metric, and scalar invariance (Kline, 2016).

Finally, Clark and Watson (2016, 2019; 2024) discussed convergent and discriminant validity, suggesting that convergent validity is more pertinent when using different methods to assess the same construct (e.g., multi-informants), whereas in a study like ours, relying on a single informant, discriminant validity is more appropriate. To establish discriminant validity, we examined relationships with variables hypothesized to be relatively proximal outcomes. This is in line with Clark and Watson (2019)’s guidelines on “near-neighbor constructs that are known to be strongly related to one another” (p. 1422), more specifically with measures that assess distinct, albeit related, constructs (Clark & Watson, 2019). In the present case, the focus was on variables hypothesized to be relatively proximal outcomes. Predictive validity will be assessed by examining associations between the BPNSFS-PSI and measures of parents’ vitality and satisfaction with their family life. Past studies using the general BPNSFS showed that these constructs have previously been linked positively to need satisfaction and negatively to need satisfaction, though past studies relied on the general BPNSF. (e.g., Cordeiro et al., 2016; Dieleman et al., 2019; Heissel et al., 2018; Liga et al., 2020, Neubauer et al., 2022; Nishimura & Suzuki, 2016). We expected to replicate these links in the present context, where our contextual measure of need satisfaction and frustration would be predictably tied to parental overall and familial functioning. Given past research on general and contextualized versions of the BPNSFS that supported its psychometric qualities (see van der Kaap-Deeder et al., 2020), we expected the BPNSFS-PSI to show similarly strong properties.

## Method

### Transparency and Openness

We report how we determined our sample size, all data exclusions (if any), all manipulations, and all measures in the study, and we followed Journal Article Reporting Standards (Kazak, 2018). Data and study materials are available upon request to the first author (it was not deposited on the repository because of the requirements of our institution’s ethics committee; *approval # masked*). This study’s design and its analysis were not pre-registered. We report how we determined our sample size, all data exclusions, all manipulations, and all measures in the study.

### Participants and Procedure

This study used two independent samples of parents whose child attended primary school at the time of data collection. Both samples are part of larger data collections.

#### Sample 1: Convenience Sample

Participants were recruited through parents’ committees from school boards across [*region masked for blind review*] as well as via social media outlets of partner organizations in the education sector. Parents received an invitation to fill out an online questionnaire on their needs in the context of their involvement in their child’s schooling. Interested parents could enroll in the study on a secure website by providing their contact information (743 families enrolled, for the potential of 1,350 parents). To participate in the online survey study, mothers and fathers needed to have a child in primary school. The sample size was determined by the number of families reached during this period. This larger study began prior to the COVID pandemic. In the present study, we are using data from Time 2 (T2) that was collected in January 2021, which corresponds to the beginning of the second school term (out of three) of primary schooling in the [*masked*] school system. Participants were asked to refer to their child attending primary school when answering questions on their school involvement. If they had more than one child in primary school, they were asked to answer in reference to their youngest child. All participants received a gift card for a national bookstore chain after each data wave.

A total of 933 parents (333 fathers, 594 mothers, 6 others, e.g., legal guardian, grandparent) from 644 families were included in Sample 1. Their average age was 41.36 (*SD* = 5.24; range = 27–76) and most participants spoke French at home (93%), obtained a college diploma or more (85%), held a full-time job (82%), and were in a stable union (87%; e.g., married, civil union). On average, 64% of the families had an annual family income of 100,000 [*currency masked*] or more, which was close to the annual family income in [*masked*] at the time of data collection (i.e., 98,690 [*currency masked*]; [*masked source*]). Target children (51% boys) were in Grade 1 (5%), 2 (26%), 3 (23%), 4 (19%), 5 (13%), and 6 (10%). School levels are grouped into three cycles composed of two academic years. About a third of children received a diagnosis for (26%) or were suspected of (16%) having special needs (e.g., attention problems, learning difficulties, emotional difficulties, giftedness). Most children lived with both their parents (84%).

#### Sample 2: Stratified Sample

Data came from a larger longitudinal study on parental needs in the context of their involvement in their child’s schooling. The [*masked*] Ministry of Education provided a random sample of primary school students who were in first or second grade in the 2020 to 2021 school year. The sample was stratified based on the school’s socioeconomic status. It also oversampled students with special needs (i.e., those who were identified by the school system as handicapped students or students with adjustment or learning difficulties) so that they constituted half of the sample. Of the 5,600 families for whom the Ministry provided contact information, 2,991 families could be reached by phone and 1,750 agreed to participate in the study, for a total of 2,218 parents agreeing to receive an online questionnaire, which was hosted on a secure university server (paper format available upon request). Sample size was determined by the number of families reached during this period. Data collection occurred twice per school year (Fall and Spring) and here, data from T2 was used (but for a subset of the sample [*N* = 921], T1 data was used to evaluate test–retest reliability). Participants received a gift card for a national bookstore chain for completing each data wave.

Sample 2 thus included 1,189 participants (899 mothers, 272 fathers, 18 others, e.g., legal guardian, grandparent) from 1,033 families, whose child was in first (55%) or second grade (45%) of primary schooling. Parents’ average age was 38.22 (*SD* = 5.53; range = 24–65) and most participants spoke [*language masked*] at home (91%), obtained a college diploma or more (69%), held a full-time job (72%), and were in a stable union (81%; e.g., married, civil union). On average, 60% of the families had an annual family income of 75,000 [*currency masked*] or more, with 41% reporting an annual income of 100,000$ or more—the average annual family income in [*masked*] at the time of data collection was 98,690 [*currency masked*] (*masked source*). Target children (63% boys) typically lived with both their parents (77%) and about 43% had a formal diagnosis regarding a special need (e.g., dyslexia, attention deficit disorder, giftedness, autism spectrum disorder) and 22% were suspected of having a diagnosable condition or were waiting for a formal diagnosis.

### Measures

#### Adaptation of Items

Because no adaptation of the BPNSFS was contextualized to parental needs during their involvement in their child’s schooling, we gathered items from (a) the general French version of the scale (24 items; Chevrier & Lennegrand, 2021) and (b) a short Flemish version adapted to maternal needs during the transition to motherhood (12 items; Brenning et al., 2019), which were translated into French. Because the items from Brenning et al. (2019) were contextualized to parenting—albeit to a distinct parenting domain (infant caregiving)—we began by adapting these to the context of school involvement. Since it only had 12 items, we included all of these and added 12 more items from the general French version and adapted these to the schooling context, when necessary. We also added the context phrase “In the tasks related to my involvement in my child’s schooling …” to encourage parents to focus on the schooling context. We mentioned that depending on their child’s age, this may refer to a variety of school-related tasks (e.g., helping the child with their homework, talking to them about school, supervising the completion of their schoolwork; see Table 1). Content validity was assessed by having three experts on parenting and SDT evaluate all items.

**Table 1.** Items of the BPNSFS-PSI.

Instructions
*The next questions pertain to your involvement in your child’s schooling. Depending on your child’s age, this may refer to a variety of school-related tasks. For example, helping your child with their homework, talking to them about school or supervising the completion of their schoolwork are all tasks related to school support.* *The following statements relate to feelings you may or may not have about your involvement in your child’s schooling. For each statement, indicate the extent to which it is true for you.*
In the tasks related to my involvement in my child’s schooling …
*Autonomy Satisfaction*
1. I feel like I’m doing things that really interest me.^a^ *J’ai le sentiment de faire des choses qui m’intéressent vraiment.*
2. I feel like I can make choices and be free in what I do.^b^ *J’ai le sentiment de pouvoir faire des choix et d’être libre dans ce que je fais.*
3. I feel like I can make decisions about how to do things.^b^ *J’ai le sentiment que je peux prendre des décisions sur les façons de faire.*
4. I feel like I can do things my way.^a^ *J’ai le sentiment de pouvoir faire les choses à ma manière.*
*Competence Satisfaction*
5. I feel competent in what I do.^a^ *Je me sens compétent dans ce que je fais.*
6. I am confident that I can do things right.^a^ *Je suis confiant dans le fait que je peux bien faire les choses.*
7. I feel that I have the ability to support my child well.^b^ *Je sens que j’ai la capacité de bien l’accompagner.*
8. I feel that I can help my child well with tasks that they find more difficult.^b^ *J’ai le sentiment de pouvoir l’aider efficacement dans des tâches qu’il trouve plus difficiles.*
*Relatedness Satisfaction*
9. I feel close and connected to my child.^a^ *Je me sens proche et liéà mon enfant.*
10. I feel affection for my child.^a^ *Je ressens de l’affection pour mon enfant.*
11. I feel that we are having a good time together.^b^ *Je sens que nous passons un bon moment ensemble.*
12. I feel like we can count on each other.^b^ *Je sens qu’on peut compter l’un sur l’autre.*
*Autonomy Frustration*
13. For most of the things I do, I feel like “I have to do this.”^a^ *Pour la plupart des choses que je fais, j’ai l’impression que « je dois le faire ».*
14. I feel compelled to do a lot of things that I wouldn’t choose to do.^a^ *Je me sens obligé de faire beaucoup de choses que je ne choisirais pas de faire.*
15. I feel pressured to do too many things.^b^ *Je me sens contraint de faire beaucoup trop de choses.*
16. I feel that each of the tasks to be done is a chain of obligations.^b^ *J’ai l’impression que chacune des tâches à faire est un enchaînement d’obligations.*
*Competence Frustration*
17. I feel insecure about my abilities.^a^ *Je ne me sens pas certain de mes compétences.*
18. I am disappointed with the way I manage to support my child.^a^ *Je suis déçu de la façon dont j’arrive à accompagner mon enfant.*
19. I have serious doubts about my ability to support my child well.^b^ *J’ai de sérieux doutes sur ma capacitéà bien accompagner mon enfant.*
20. I feel like a bad parent because of how difficult it is to support them.^b^ *J’ai le sentiment d’être un mauvais parent à cause des difficultés que j’ai à bien l’accompagner.*
*Relatedness Frustration*
21. I feel that my relationship with my child is cold and distant.^a^ *J’ai le sentiment que la relation que j’ai avec mon enfant est froide et distante.*
22. I feel distance between my child and me.^a^ *Je ressens un sentiment de distance entre mon enfant et moi.*
23. I feel like we are drifting apart.^b^ *Je sens que nous nous éloignons l’un de l’autre.*
24. I feel like my child doesn’t like me.^b^ *J’ai l’impression que mon enfant ne m’apprécie pas.*

*Note*. Items were translated into English using the back-translation procedure (Vallerand, 1989), and were then reviewed by a native English speaker.

a Item adapted from Brenning et al. (2019); ^b^Item adapted from Chevrier and Lannegrand (2021).

Rated on a 5-point scale where 1 = *Completely false*, 2 = *Somewhat false*, 3 = *Neutral*, 4 = *Somewhat true*, and 5 = *Completely true*.

BPNSFS-PSI = Basic Psychological Need Satisfaction and Frustration Scale-Parents’ School Involvement.

The final 24 items adapted to parents’ involvement in their child’s schooling are presented in Table 1. Participants were asked to indicate the extent to which each item represented how they felt about their involvement in their child’s schooling (i.e., how true these were for them), using a 5-point Likert scale that ranged from 1 (*completely false*) to 5 (*completely true*). The adapted scale included six subscales (4 items each): autonomy satisfaction, autonomy frustration, competence satisfaction, competence frustration, relatedness satisfaction, and relatedness frustration. The psychometric properties of the scale are presented in the Results section.

#### Satisfaction With Family Life

The Satisfaction with Life Scale (Diener et al., 1985; French translation by Blais et al., 1989) was adapted to measure how satisfied parents were with respect to their family life. Parents were asked to indicate their level of agreement with each of the five items, using a 7-point scale ranging from 1 (*do not agree at all*) to 7 (*agree very strongly*). The items were: “My relationship with my immediate family is very good,”“My immediate family and I have a stable relationship,”“My immediate family and I have a strong relationship,”“My family climate makes me happy,” and “I really feel that my immediate family and I are close.” This scale was found to be reliable (ω = .83).

#### Vitality

The Subjective Vitality Scale (Ryan & Frederick, 1997) was used to assess parents’ level of energy available to the self. This scale was rated on a 7-point scale where parents had to indicate their level of agreement with each statement (7 items; e.g., “I look forward to each new day”) from 1 (*do not agree at all*) to 7 (*agree very strongly*). Past research supported the psychometric quality of the scale, which was replicated here (ω = .87).

#### Sociodemographic Variables

Parents answered questions pertaining to themselves (e.g., gender, age, education level, occupational status), their child (e.g., age, gender, school level, diagnosis), and their family (e.g., socioeconomic status, language spoken at home, family structure).

### Statistical Analyses

#### Model Estimation

Measurement models were estimated with Mplus (version 8.8; Muthén & Muthén, 1998–2022) under robust maximum likelihood estimator, which is suggested when testing SEM models with continuous indicators (Swami et al., 2023). This estimator uses the Mplus design-based correction of standard errors (Asparouhov, 2005) and yields standard errors and fit indices that are robust to non-normality. The adequacy of model fit was estimated with the comparative fit index (CFI; Bentler, 1990), the Tucker-Lewis index (TLI; also known as the Bentler-Bonett non-normed fit index; Bentler & Bonett, 1980), and the root mean squared error of approximation (RMSEA). Values greater than .90 for the CFI/TLI and lower than .08 for the RMSEA are considered to indicate adequate fit to the data, whereas values above .95 for CFI/TLI and below .05 for the RMSEA indicate excellent model fit (e.g., Hu & Bentler, 1999; Marsh et al., 2005). Because some of the parents belonged to the same family unit, the interdependent nature of the data was controlled for by using family as a cluster and the TYPE = COMPLEX command in Mplus.

#### Missing Data

In both samples, the percentage of missing data was rather low, ranging from 0% to 1.5%. Missing data was statistically handled using full information maximum likelihood (FIML) estimation (Enders, 2022; Graham, 2012; Kline, 2016).

#### Testing Factorial Structure

The factor structure of the scale was examined by testing the 17 models presented above. Nonhierarchical models included single factor (Model 1), 2-factor (in CFA [Model 2] and ESEM [Model 3]), 3-factor (autonomy, competence, relatedness; in CFA [Model 4] and ESEM [Model 5]), and 6-factor (in CFA [Model 6] and ESEM [Model 7]) solutions. Hierarchical models were estimated using bifactor parametrization (Morin et al., 2016) and utilizing the same models presented above while adding global factors: (a) 2 specific factors and one global factor (in CFA [Model 8] and ESEM [Model 9]); (b) 3 specific factors with one global factor (in CFA [Model 10] and ESEM [Model 11]) and with two global factors (in CFA [Model 12] and ESEM [Model 13]); (c) 6 specific factors with one global factor (in CFA [Model 14] and ESEM [Model 15]) and with two global factors (in CFA [Model 16] and ESEM [Model 17]). CFA and ESEM models were compared and, if equally satisfying, the CFA solution was retained for parsimony (Marsh et al., 2014).

#### Estimating Internal Consistency

To evaluate the internal consistency of all subscales of the BPNSFS-PSI, we calculated McDonald omega values, which are preferable to Cronbach alpha (McNeish, 2018; Hayes & Coutts, 2020).

#### Invariance Analyses

Once an optimal measurement model was established for the merged samples, invariance was tested to ensure the questionnaire behaved in a similar fashion for each sample, for mothers and fathers, and as a function of their child’s school cycle (in [*masked*] primary education, school levels are grouped into 3 cycles). Three types of measurement invariance were tested (Kline, 2016; Putnick & Bornstein, 2016): (a) configural invariance (i.e., whether the number of factors and corresponding items remain the same across groups); (b) metric invariance (i.e., whether factor loadings and latent factors are stable across groups); and (c) scalar invariance (i.e., whether intercepts of latent factors are invariant across groups). When comparing solutions, we used the Satorra-Bentler scaled chi-square difference test (χ^2^_SB_; Satorra & Bentler, 1994), where a statistically nonsignificant coefficient suggested that two nested models were invariant. The difference between two nested models was also considered to be meaningful if their CFI and SRMR values differed by 0.010 or more and if RMSEA values differed by 0.015 or more (Chen, 2007). Nonhierarchical models were compared using Akaike’s Information Criterion (AIC) such that the smallest AIC value represented the best-fitting model (Kline, 2016).

#### Interpretation of Results

In line with the recommendation of Wilkinson and the APA Task Force on Statistical Inference (1999) and aligned with what has been labeled the *statistics reform* (Kline, 2013; or *new statistics*; Cumming & Calin-Jageman, 2016), parameter estimates were interpreted using effect size estimates (e.g., η^2^, β, *R*^2^) rather than the results of null hypothesis significance testing (i.e., *p* values). Interpretations thus focused on effect sizes (e.g., *r* and β ≥ .10) and proportions of explained variance, regardless of *p* values. For mean comparisons, interpretations relied on partial eta squared (η^2^), which estimates the proportion of variance in a variable (e.g., competence satisfaction) explained by group variables (e.g., child difficulties), and whether 95% confidence intervals (CIs) for group means overlapped.

## Results

### Preliminary Analyses

Descriptive statistics (i.e., correlations, means, standard deviations) are reported in Table 2. Results show that the needs were correlated with each other in the expected directions. Specifically, measures of satisfaction for all three needs were positively associated with each other and negatively associated with the frustration of all three needs, while measures for the frustration of all three needs were positively associated with each other. The strength of the coefficients indicates that these measures were not redundant. Their links with sociodemographic variables were negligible. When inspecting the means for each measure, four main conclusions were drawn: (a) parents typically reported strong satisfaction for their competence and relatedness needs and moderate autonomy satisfaction; (b) they also reported weak frustration of their needs although autonomy frustration was moderate to low; (c) the most satisfied need appeared to be relatedness; and (d) the most frustrated need was autonomy.

**Table 2.** Correlations, Means, and Standard Deviations for all Variables (*N* = 2,035).

Variables	1	2	3	4	5	6	7	8	9	10	11
1. Autonomy satisfaction^a^	—										
2. Autonomy frustration^a^	−.37	—									
3. Competence satisfaction^a^	.55	−.36	—								
4. Competence frustration^a^	−.40	.45	−.74	—							
5. Relatedness satisfaction^a^	.60	−.27	.46	−.40	—						
6. Relatedness frustration^a^	−.34	.27	−.38	.49	−.55	—					
7. Vitality^b^	.30	−.23	.31	−.29	.25	−.17	—				
8. Satisfaction with family life^b^	.21	−.22	.26	−.27	.26	−.30		—			
9. Parent gender^c^	.07	.04	.02	−.02	.08	−.06	−.09	.04	—		
10. Parent age	−.05	−.01	.02	−.02	.02	−.02	−.01	−.05	−.25	—	
11. Child gender^d^	.04	−.05	.02	−.03	.05	−.04	.02	.07	.03	−.01	—
12. School level	−.04	−.09	.02	−.03	−.04	.05	.03	.05	−.11	.20	.09
*M*	3.92	2.71	4.21	1.72	4.52	1.30	4.35	5.97		39.14	
*SD*	0.84	1.01	0.77	0.84	0.61	0.64	1.14	0.99		5.46	
ω	.85	.88	.92	.88	.85	.93	.87	.83			

*Note*. ^a^Rated on a 5-point scale; ^b^rated on a 7-point scale; ^c^1 = *father*, 2 = *mother*; ^d^1 = *boy*, 2 = *girl*.

For coefficients whose value was *r* ≥ |.05|, *p* < .05.

### Scale Reliability

First evidence for the reliability of the BPNSFS-PSI is its internal consistency. As reported in Table 2, omega values for all subscales were satisfactory (all ωs > .80), suggesting that the measure of each need, both with respect to its satisfaction and its frustration, yielded a homogeneous pattern of response across items. Second, we examined the temporal stability of the subscales over a seven-month period. Correlations were relatively strong, showing stability without redundancy (i.e., indicating sufficient variability of the needs across time), with coefficients ranging from .52 (*relatedness frustration*) to .66 (*competence frustration*).

### Structural Validity

To establish structural validity, all 17 measurement models were estimated (see Table 3). Two hierarchical models yielded similarly high, excellent fit indices: the bifactor ESEM models with one g-factor, representing relative need satisfaction versus frustration (Model 15) and the one that included two *g*-factors, one representing need satisfaction while the other represented need frustration (Model 17). To determine which of the two models best represented the data, we first examined whether the two *g*-factors were distinct enough (i.e., discriminant validity) by inspecting their correlations in Models 12 and 16 (CFA models) and in Models 13 and 17 (ESEM). The CFA models found the correlations between the g-factors to be strong (Model 12: *r* = −.66; Model 16: *r* = −.70), suggesting low discriminant validity. Making this distinction entails a complexity that seems to outweigh its potential substantive benefits in terms of differentiated prediction of outcomes from poorly defined factors. While correlations among satisfaction and frustration *g*-factors were lowered in bifactor ESEM parameterizations (Model 13: *r* = −.05; Model 17: *r* = .03), these factors were ill-defined, as indicated by their low factor loadings (λs = .01–.86, *M* = 0.18). There is thus little evidence supporting the need to distinguish between need satisfaction and frustration g-factors. Model 15 was thus retained.

**Table 3.** Goodness-of-Fit Statistics for the Estimated Models on the BPNSFS.

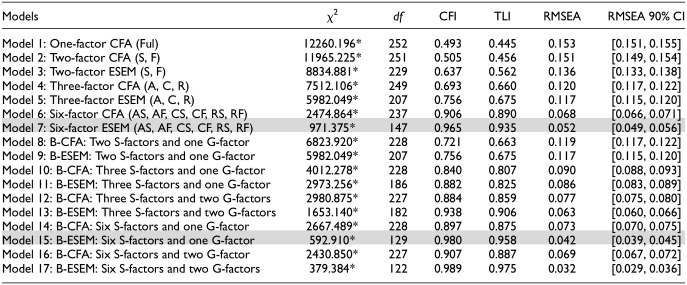

*Note*. χ^2^ = robust chi-square test of exact ﬁt; *df* = degrees of freedom; BPNSFS = Basic Psychological Need Satisfaction and Frustration Scale-Parents’ School Involvement; CFI = comparative ﬁt index; TLI = Tucker–Lewis index; RMSEA = root mean square error of approximation; 90% CI = 90% conﬁdence interval of the RMSEA; CFA = conﬁrmatory factor analysis; ESEM = exploratory structural equation modeling; B = bifactor model; Ful = global need fulﬁllment; S = need satisfaction; Fr = need frustration; A = need for autonomy; C = need for competence; R = need for relatedness; G-factor = global factor estimated as part of a bifactor model; S-factor = speciﬁc factor estimated as part of a bifactor model.

* *p* < .01. *N* = 2,035.

In this retained solution, the global factor can be interpreted as reflecting general need fulfillment while specific factors reflect specific facets of need fulfillment, over and above the general need fulfillment level. Table 4 presents the factor loadings for this retained measurement model. All items loaded in the expected direction on the global factor representing need fulfillment, with items measuring need satisfaction loading positively and frustration items loading negatively. The general factor was well-defined, as demonstrated by the strength of factor loadings (λs ≥ |.44|), although one item measuring autonomy frustration displayed a lower factor loading. The *g*-factor represented parents’ global need fulfillment, as indicated by the positive loadings from items representing autonomy, competence, and relatedness satisfaction, and the negative loadings from the items representing autonomy, competence, and relatedness frustration. For the specific factors, results also showed that the items generally loaded more strongly and positively on their target factor (see shaded cells in Table 4; λs = .36–.84) than on the other factors, except for two autonomy satisfaction items whose coefficients were weaker, suggesting that they more strongly reflected general need fulfillment than autonomy satisfaction.

**Table 4.** Factor Loadings From the Bifactor ESEM Model with One G-Factor for the BPNSFS-PSI.

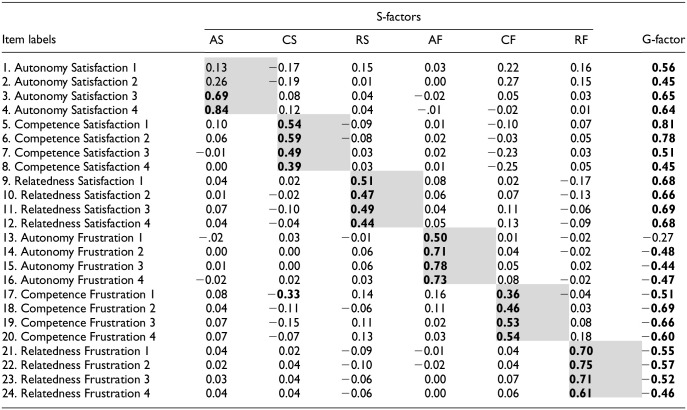

*Note*. Coefficients that are notable (λ ≥ .30) are in bold. Shaded cells represent factor loadings on their target factor. AS = autonomy satisfaction; CS = competence satisfaction; RS = relatedness satisfaction; AF = autonomy frustration; CF = competence frustration; RF = relatedness frustration.

BPNSFS-PSI = Basic Psychological Need Satisfaction and Frustration Scale-Parents’ School Involvement; ESEM = exploratory structural equation modeling.

One nonhierarchical model yielded a similarly excellent fit, namely the ESEM model with six factors (each representing a subscale of the BPNSFS-PSI; Model 7). Factor loadings are presented in Table 5; they show that each item adequately assessed its respective subscale, thereby offering stronger support for the structural validity of the scale. Specifically, the results revealed that all items loaded more strongly on their target factor (λs = .32–.99) than on other factors, with the two same autonomy satisfaction items also loading well on relatedness satisfaction. Because researchers might also want to use the BPNSFS-PSI to link specific subscales to constructs of interest (e.g., competence frustration as a predictor of parents’ difficulty in offering a structuring environment to their child) and are not focused on the role of general need fulfillment, this nonhierarchical solution is a satisfying option.

**Table 5.** Factor Loadings From the 6-Factor ESEM Model for the BPNSFS-PSI.

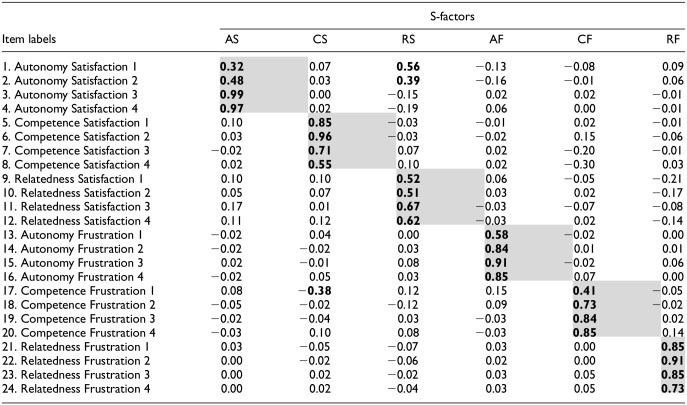

*Note*. Coefficients that are notable (λ ≥ .30) are in bold. Shaded cells represent factor loadings on their target factor. AS = autonomy satisfaction; CS = competence satisfaction; RS = relatedness satisfaction; AF = autonomy frustration; CF = competence frustration; RF = relatedness frustration.

BPNSFS-PSI = Basic Psychological Need Satisfaction and Frustration Scale-Parents’ School Involvement; ESEM = exploratory structural equation modeling.

### Testing Generalizability

**Figure 1.**
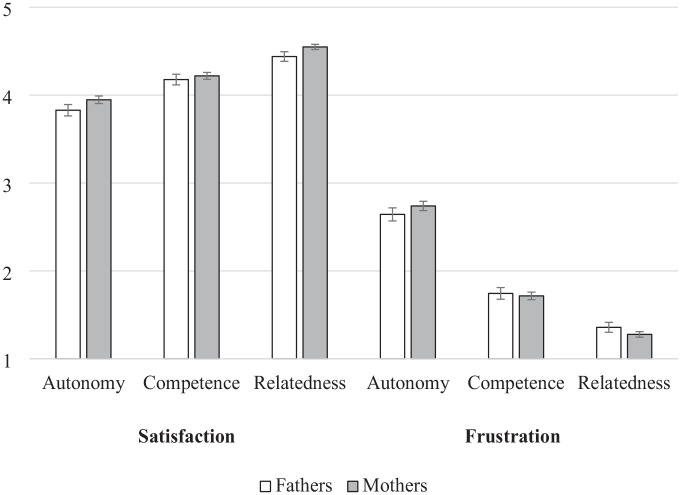
Mean Scores for Mothers’ and Fathers’ Psychological Need Satisfaction and Frustration. *Note*. Response scale varied from 1 to 5.

Using the measurement model that yielded the best solution, invariance analyses were conducted to ascertain that the BPNSFS-PSI behaved equally well across samples, parent gender, and child school cycles. As reported in Table 6, invariance analyses provided strong support for configural, metric, and scalar invariance (a) between Sample 1 and Sample 2 participants, (b) between mothers and fathers, and (c) for parents whose child was in first, second, and third cycles of primary school. Figure 1 presents mothers’ and fathers’ levels of satisfaction and frustration with each need. Results indicate that mothers reported slightly higher autonomy and relatedness satisfaction than fathers did, whose CIs did not overlap, although the size of these differences was negligible (*d*s < 0.01). Their levels of competence satisfaction were similar, as per their overlapping CIs. For measures of need frustration, all CIs overlapped, indicating no meaningful difference between mothers and fathers.

**Table 6.** Goodness-of-Fit Indices of the Multigroup Invariance Testing.

Description	χ^2^ (*df*)	SCF	CFI	SRMR	RMSEA	90% CI	CM	*Δ*χ_SB_^2^ (*df*)	ΔCFI	ΔSRMR	ΔRMSEA	Decision
Across Samples (Sample 1 vs. Sample 2)												
M1. Configural invariance	741.45 (258)*	1.26	0.980	0.012	0.043	[0.039, 0.047]	—	—	—	—	—	—
M2. Metric invariance	827.81 (377)*	1.35	0.981	0.021	0.034	[0.031, 0.037]	M1	118.63 (119)	+0.001	+0.009	−0.009	Accept
M3. Scalar invariance	856.82 (394)*	1.33	0.981	0.022	0.034	[0.031, 0.037]	M2	26.87 (17)	+0.000	+0.001	+0.000	Accept
Across Parents’ Gender (Men vs. Women)												
M1. Configural invariance	738.37 (258)*	1.23	0.980	0.012	0.043	[0.039, 0.046]	—	—	—	—	—	—
M2. Metric invariance	857.65 (377)*	1.33	0.980	0.031	0.035	[0.032, 0.039]	M1	148.58 (119)*	+0.000	+0.019	−0.018	Accept
M3. Scalar invariance	931.93 (394)*	1.30	0.978	0.032	0.037	[0.034, 0.040]	M2	88.54 (17)*	−0.002	+0.001	+0.002	Accept
Across Child’s School Cycle (First vs. Second vs. Third Cycle)												
M1. Configural invariance	1022.28 (387)*	1.12	0.975	0.013	0.049	[0.046, 0.053]	—	—	—	—	—	—
M2. Metric invariance	1232.69 (625)*	1.26	0.976	0.032	0.038	[0.035, 0.041]	M1	271.51 (238)	+0.001	+0.019	−0.011	Accept
M3. Scalar invariance	1274.89 (659)*	1.21	0.976	0.033	0.037	[0.034, 0.040]	M2	36.58 (34)	+0.000	+0.001	−0.001	Accept

*Note. df* = degrees of freedom; SCF = scalar correction factor; CFI = comparative fit index; RMSEA = root mean square error of approximation; 90% CI = 90% confidence interval; CM = comparison model; Δ = change in fit relative to the CM; *χ*_SB_^2^ *=* Satorra-Bentler Scaled Chi Square.

* *p* < .05.

### Predictive Validity

The final step involved examining the links between the subscales of the BPNSFS-PSI and other related constructs. Measures of parents’ vitality and satisfaction with family life were entered in Model 7 (nonhierarchical modeling of the 6 needs factors) and Model 15 (bifactor modeling) to estimate their links with satisfaction and frustration of each need, as well as with need fulfillment more globally. The coefficients presented in Table 7 show that the satisfaction of autonomy, competence, and relatedness needs were positively associated with both satisfaction with family life and vitality, while the opposite pattern was found for the frustration of these needs. The strength of these associations varied from moderately weak (for autonomy satisfaction) to moderate (for competence and relatedness frustration). Results with Model 15 also indicate that global need fulfillment was positively associated with these two correlates, with a moderate effect size for satisfaction with family life and a moderately strong effect size for vitality. Together, these findings offer support to the construct validity of the BPNSFS-PSI.

**Table 7.** Construct Validity for the B-ESEM Six S-Factors and One G-Factor and for the Six-Factor ESEM.

	Satisfaction with family life		Vitality	
Psychological Needs	*r*_B-ESEM_	*r*_ESEM_	*r*_B-ESEM_	*r*_ESEM_
Autonomy satisfaction	−.06	**.18**	.02	**.27**
Autonomy frustration	−.07	**−.23**	−.05	**−.26**
Competence satisfaction	.03	**.26**	.02	**.33**
Competence frustration	−.02	**−.29**	−.01	**−.31**
Relatedness satisfaction	.01	**.22**	−.03	**.25**
Relatedness frustration	**−.13**	**−.29**	.06	**−.17**
G-factor_Need Fulfilment_	**.34**	—	**.40**	—

*Note. r*s Are all statistically significant at *p* < .001. Coefficients in bold represent effect sizes that are at least small in magnitude (*r*s ≥ .10).

*N* = 2,035.

Fit indices for ESEM parametrization: χ^2^ (476) = 2195.55, *p* < .01, CFI = 0.96, TLI = 0.94, RMSEA = 0.04 [0.040, 0.044], SRMR = 0.03.

Fit indices for B-ESEM parametrization: χ^2^ (456) = 1756.52, *p* < .01, CFI = 0.97, TLI = 0.96, RMSEA = 0.04 [0.036, 0.039], SRMR = 0.03.

## Discussion

This study adapted the BPNSFS to the context of PSI. The findings of this multi-sample study supported the psychometric qualities of the BPNSFS-PSI for measuring the satisfaction and the frustration of parents’ psychological needs for autonomy, competence, and relatedness when they are involved with their child’s schooling. Two measurement models offered an excellent fit: one hierarchical (bifactor ESEM model with six specific factors and one general factor) and one nonhierarchical (6-factor ESEM model). These findings suggest that the six subscales assess distinct constructs that are non-redundant but also that their items can be grouped under a general factor representing need fulfillment. Each subscale was found to be reliable and to correlate with each other in the expected direction (i.e., satisfaction subscales positively correlated with each other and negatively correlated with frustration subscales, which themselves correlated positively together). The BPNSFS-PSI was also demonstrated to be invariant as a function of samples, parent gender, and child school cycle. Finally, construct validity of the scale was established by demonstrating positive correlations between autonomy, competence, and relatedness satisfaction and measures of parents’ vitality and satisfaction with family life, as well as with the negative correlations between these two outcomes and the frustration of the three needs.

### Implications

The central contribution of this study is the provision of a psychometrically sound measure to assess parents’ need satisfaction and frustration in the context of school involvement. Being able to assess both the satisfaction and frustration of parents’ psychological needs can help understand the unfolding of important outcomes such as positive parental behaviors (e.g., autonomy support) using the need satisfaction subscales, and negative parental behaviors (e.g., control) using the frustration subscales when parents engage in their child’s schooling. This is important, given that parental involvement has been found to be crucial for children’s learning capabilities (Desforges & Abouchaar, 2003; Hornby & Lafaele, 2011). However, from an SDT perspective, predicting the consequences of this involvement for parents and children is necessarily tied to the quality of their involvement. For parents to engage in high-quality school involvement, satisfaction of their psychological needs is regarded to be crucial, in line with previous work pertaining to parents’ general needs and behaviors (e.g., Costa et al., 2019; Slobodin et al., 2020; van der Kaap-Deeder et al., 2015). The BPNSFS-PSI will therefore contribute to future research in this important domain by offering an appropriate measure of parental needs.

Our results also demonstrated the structural validity of the BPNSFS-PSI via both nonhierarchical and hierarchical six-factor solutions. Depending on researchers’ and practitioners’ needs, it is possible to use this scale with the aim of distinguishing each parental need both with respect to their satisfaction and frustration. They could also choose one specific subscale for testing a targeted hypothesis (e.g., Is parental competence in their school involvement different depending on their education level?). Results of the nonhierarchical model support such use of the BPNSFS-PSI. In contrast, researchers might want to model items to extract a global factor (i.e., need fulfillment) to be used in a complex model, for instance by saving the factor score (Skrondal & Laake, 2001), or in conjunction with the specific factors to test how global and specific factors each relate to constructs of interest.

Relatedly, the present findings align with those of previous studies that showed strong support for the modeling of psychological needs using bifactor models in contexts such as exercise (Rodrigues et al., 2021), physical education (Garn et al., 2019), university studies (Gillet et al., 2019), driving behaviors (Stiegemeier et al., 2024), or work (Sánchez-Oliva et al., 2017). While bifactor models were criticized in the past, their use in the SDT literature is expanding (see Howard, 2023). They entail benefits such as allowing for simultaneous modeling of a general factor that represents the global level of need fulfillment together with specific factors representing the satisfaction and frustration of autonomy, competence, and relatedness needs. More importantly, this is also in line with the conceptual background of the needs. That is, as shown in previous research (Ratelle & Duchesne, 2014; see Vansteenkiste et al., 2023), the satisfaction of one need often co-occurs with the satisfaction of the other two needs. Thus, in practice, the satisfaction of all three needs will often go hand in hand, even though they are theoretically distinct constructs.

Collectively, the results of this study show the BPNSFS-PSI to be an appropriate measure for parents’ satisfaction and frustration of their needs for autonomy, competence, and relatedness while being involved in their child’s schooling, like when they help the child with their homework, talk with them about school, or supervise the completion of their schoolwork. This scale can be used by parents to identify why they sometimes struggle with these tasks (e.g., *Do I feel coerced into doing them?*) and guide them toward reducing sources of such frustration (e.g., *Am I putting too much pressure on myself?*) The BPNSFS-PSI can also be implemented in the context of family-school partnerships for parents whose school involvement is suboptimal to identify which mechanisms can be targeted (e.g., supporting their competence through guidance and scaffolding) to improve the quality of their involvement in their child’s schooling.

### Strengths, Limitations, and Future Directions

This study had important strengths, such as the use of two independent samples—of which one is a representative stratified sample—the size of these samples (more than 2,000 participants combined), sophisticated statistical modeling, and the demonstration that the BPNSFS-PSI was invariant across samples, parent gender, and child school cycle. Even though most participants were mothers, a substantial part of the participants consisted of fathers. This is important given that most of the parenting research has solely included mothers, limiting our understanding of fathers’ unique roles in their child’s development.

Nonetheless, there are important limitations that must be considered when interpreting the results. First, the study relied on a single informant, which could have increased biases due to shared method variance when estimating construct validity. Future studies should examine the links between this measure and other constructs evaluated by other informants (e.g., triangulation with child-reported autonomy support). Furthermore, the sample was characterized by low diversity with respect to parents’ ethnocultural characteristics, socioeconomic background, and sexual orientation. The PBNSFS-PSI should be examined in more diverse samples that include parents who are not heteronormative as well as parents from minority cultural groups, and more socioeconomically disadvantaged contexts. With regards to scales, there appeared to be ceiling effects for parents’ levels of need satisfaction, suggesting that parents who agreed to participate in our study were generally feeling autonomous, competent, and positively related when engaging in their child’s schooling. It is possible that the scale behaved differently in parents who experience more difficulties in that domain, for instance, by recruiting them via community organizations dedicated to supporting parent-school collaboration. However, it must be noted that about 50% of the parents in our second sample consisted of parents of students with special needs, indicating the applicability and relevance of the PBNSFS-PSI in this subsample. Finally, cross-sectional data was used such that longitudinal invariance was not tested. We chose not to consider T1 data from Sample 1 because it occurred prior to the pandemic, or T1 data from Sample 2, which took place at the beginning of the school year, such that parents’ psychological needs might not have been affected much. Future studies should test the longitudinal invariance of the PBNSFS-PSI.

In future research, it will be important to further test the construct validity of the BPNSFS-PSI by examining its link with parents’ motivations toward the school involvement, which should be proximally predicted by their psychological need satisfaction—which should facilitate autonomous motivation toward their school involvement—and frustration—which should engender controlled motivation and amotivation toward their school involvement. Similarly, future studies should focus on the relation between parents’ needs in the context of school involvement and the quality of this involvement, by assessing the dimensions of parental autonomy support, structure (or competence support), and warmth (or relatedness support). Importantly, our focus should not only be on how parental need satisfaction can facilitate their need supportive behaviors toward their child but also how their need frustration can drive them toward need-thwarting behaviors (i.e., being controlling, chaotic, and rejecting toward their child). Finally, the use of a longitudinal design will not only allow for testing the longitudinal invariance of the BPNSFS-PSI but also for examining developmental patterns of need satisfaction and frustration during PSI over time.

## Conclusion

Given the benefits of high-quality parental involvement for their child’s schooling, the importance of individuals’ psychological needs for autonomy, competence, and relatedness, and the prior lack of a scale to assess parents’ needs in the context of school involvement, this research set out to develop and validate an adaptation of the BPNSFS for this specific context. Our findings showed that the BPNSFS-PSI displays adequate psychometric properties among two large samples, which can inform and foster future research on this important topic. Assessing parental needs will be helpful for identifying important mechanisms underlying suboptimal practices they adopt in their school involvement (e.g., disengagement, controlling behaviors toward their child during homework). Such an endeavor will help in designing efficient parental intervention programs, such as the Parent Check-In (Grolnick et al., 2021) or the How-To program (Mageau et al., 2022), which teach parents to adopt behaviors that support the satisfaction of their child’s psychological needs and refrain from using need thwarting behaviors.
